# Supplementary material for: Younger Dryas ice margin retreat triggered by ocean surface warming in central-eastern Baffin Bay
Source: Nat Commun. 2017 Oct 18;8:1017. doi: 10.1038/s41467-017-01155-6 (PMC5715068; doi:10.1038/s41467-017-01155-6)
Supplement: Supplementary file 1 — Supplementary Information [file 41467_2017_1155_MOESM1_ESM.pdf]

Supplementary Note 1 | Modern surface hydrography based on factor analysis

The Q-mode factor analysis applied to the North-Atlantic surface sample data set[1] reveals eight factors that together account for more than 95% of the total variance in the calibration data set. On the basis of their indicative species the assemblages are interpreted as: the Marginal Ice Zone assemblage, the Arctic Water assemblage, the Greenland Arctic Water assemblage, the East-West Greenland Current assemblage, the Transitional Water assemblage, the Subarctic Water assemblage, the North-Atlantic Current assemblage and the Norwegian Atlantic Current assemblage (Supplementary Fig. 1). Our analysis yielded results nearly identical to previous studies[2, 3] that have demonstrated a close association between the spatial distributions of factor loadings and a modern spatial pattern of distinctly different surface water masses in the North Atlantic basin.

Closer investigation of the 12 surface samples in the North Atlantic calibration data set, which were collected from the Baffin Bay region (65-75°N), reveals that the modern Baffin Bay and the Labrador Sea feature a regional dominance of the Arctic Water and the Marginal Ice Zone assemblages, with a minor contribution of the East-West Greenland Current and the Greenland Arctic Water assemblages. They show a pronounced southward shift in the maximum loading of the Marginal Ice Zone and the East-West Greenland Current factors (Supplementary Table 1). These samples also show high communalities, except for samples 173 and 178. The same assemblages dominate core SL 170, as shown in Supplementary Fig. 2.

Supplementary Note 2 | Comparison of modern and downcore diatom assemblages

In order to assess the similarity between modern surface sediment and downcore assemblages, we compared the relative abundances of the 14 most common species in core SL 170 (Supplementary Fig. 3) with the relative abundances of those 14 species in the 12 surface samples from Baffin Bay included in the North Atlantic calibration data set (Supplementary Fig. 4). In the surface samples these 14 species compose 82.4-96.2% (avg. 89.2%) of the total counts per sample indicating that the species composition has remained largely the same from deglaciation to modern day and only the relative abundances of the species have changed. The comparison between these two diatom records shows that the dominating species *Thalassiosira antarctica* var. *borealis* spore and *Fragilariopsis oceanica* have very similar abundances in the modern and downcore records. The same applies to most of the 14 species, with a few notable differences: *Coscinodiscus marginatus* is clearly more abundant in the downcore record than in modern samples, whereas *Actinocyclus curvatulus*, *Thalassiosira gravida* and *Thalassiosira trifulta* are more common in the modern data set compared to downcore data.

Supplementary Note 3 | Chronology

Marine radiocarbon chronologies at high latitudes have uncertainties which are largely related to the poor constraining of the local ^{14}C reservoir correction age (ΔR) through times. Here we have applied a ΔR age of 140±35 years[4] that has been estimated from historical (pre-bomb) samples used in several studies in the Disko Bay region[5-11]. Previous marine studies from Baffin Bay have applied ΔR ages ranging from 0 years[12, 13] up to 400 years[14]. Due to the wide range of previously used ΔR ages and the significant impact the applied ΔR can have on the chronology, we have run several age-depth models varying only the ΔR age (Supplementary Fig. 5) and varying the ΔR age within the YD period (Supplementary Fig. 6). In addition to the chronology used in this study, we created five age-depth models using constant ΔR ages of 0±0 years, 200±100 years, 400±100 years, 1000±100 years and variable ΔR values between planktonic foraminifera (140±35 years) and benthic foraminifera (400±50 years). Further, we created three age-depth models where ΔR is set at 140±35 years for the early Holocene and for the Bølling-Allerød period, and where it varies within the YD (200±100 years, 400±100 years and 1000±100 years). The influence of the varying ΔR ages in the created models was assessed against the aSST-record and against the timing of the detrital carbonate layers in core SL 170[15]. In addition to the detrital carbonate events, no independent time markers, such as tephras, were found in the core in order to test the reservoir effect. On the other hand, tephras found in high-latitude marine sediment cores around Greenland are often considered to be ice rafted after being stored in glacier ice for an extended time and thus cannot be considered to represent primary atmospheric fallout of tephras[6, 16, 17]. Despite the lack of alternative data sources to constrain ΔR, Jackson *et al.*[15] pointed out that the difference between the ^{14}C ages of planktonic and benthic foraminifera from the same sample depths (74-76 cm and 399-402 cm) suggest that the offset between surface and deeper water is not larger than a few centuries (127-347 years).

Varying the constant ΔR correction age has a clear impact on the chronology changing the inferred ages by several hundred years (Supplementary Fig. 5 and Supplementary Table 2). As expected, only setting the ΔR to 0±0 years shifts our chronology towards slightly older ages, whereas for all the other used ΔR values, the opposite is true (Supplementary Fig. 5). However, the fact that our inferred events become more recent as we increase the ΔR age does not change the main results and interpretations presented in this study, since the onset of the aSST increase (13.4 kyr BP) still remains within the boundaries of the uncertainty (95 % confidence interval) of the used chronology. It is also noteworthy that for all the chronologies constructed using the range of ΔR values of 0±0 years, 200±100 years, 400±100 years or the variable ΔR, the YD interval in the study area still shows warmer aSSTs (Supplementary Fig. 5). A substantial change in the interpretation of the presented results would have required the application of a much larger ΔR of 1000±100 years. The chronology based on the ΔR of 1000±100 years shows the initial warming of the surface waters occurring relatively late at ca. 12.5 kyr BP and the warm aSST period mainly post-dates the YD interval (Supplementary

Fig. 5). Moreover, the very high ΔR age shifts the timing of the Baffin Bay Detrital Carbonate events BBDC0 and BBDC1 in the core. These detrital carbonate rich layers are linked with the North Atlantic Heinrich events[18] which are associated with massive meltwater and iceberg discharge[19-22]. Based on our chosen chronology (with a ΔR of 140±35 years), the BBDC-events in core SL 170 have a timing of ca. 14.2-13.7 kyr BP and ca. 12.7-11 kyr BP and are found to be synchronous across the Baffin Bay[15]. The timing of these layers based on our chosen age model corresponds well with previously identified BBDC1[19-22] and BBDC0[6, 21-24] events. The use of the ΔR = 1000±100 years would therefore yield a significant offset in the timing of the BBDC-events in SL 170 relative to the previously identified larger scale BBDC-events. Interpreting the older DC-layer as a signature of the BBDC0 event would have required applying an even higher value of ΔR, which in turn would then have shifted the younger DC-layer well into the Holocene where it would post-date the main deglaciation in the Disko Bay region[25, 26]. We thus conclude that, for the area and the time period considered, the use of very high ΔR values (1000±100 years) is not supported by the available data.

Varying the ΔR correction age only for the YD period while using the ΔR of 140±35 years for the early Holocene and for the Bølling-Allerød period does not change the interpretations of our results. The warmer sea surface temperatures still occur within the YD; even in the most extreme (ΔR = 1000 years±100) experiment only the end of the warmer sequence is shifted into the early Holocene (Supplementary Fig. 6). The rapid advance and collapse of the Jakobshavn Isbræ would still occur under warmer ocean conditions. Increasing the ΔR age up to 1000 years for the YD period causes age reversals at the transitions between the time periods, thus using the combination of the reservoir corrections 140±35 years and 1000±100 years can be rejected.

We postulate that the radiocarbon chronology presented here is solid and reliable based on the following points: (1) the duplicate samples from the same sample interval resulted in similar ages (Supplementary Table 2), (2) planktonic and benthic foraminifera ages from the same sample agree relatively well with each other, i.e. the offset is not more than few centuries (Supplementary Table 2), (3) the application of the larger ΔR age, the variable ΔR ages or the varying ΔR ages within the YD period do not significantly shift the timing of the warmer aSST interval discussed in this study, and (4) the presence of the detrital carbonate layers consolidates the SL 170 radiocarbon chronology by acting as an independent time marker and establishing the link to other marine sediment cores in Baffin Bay.

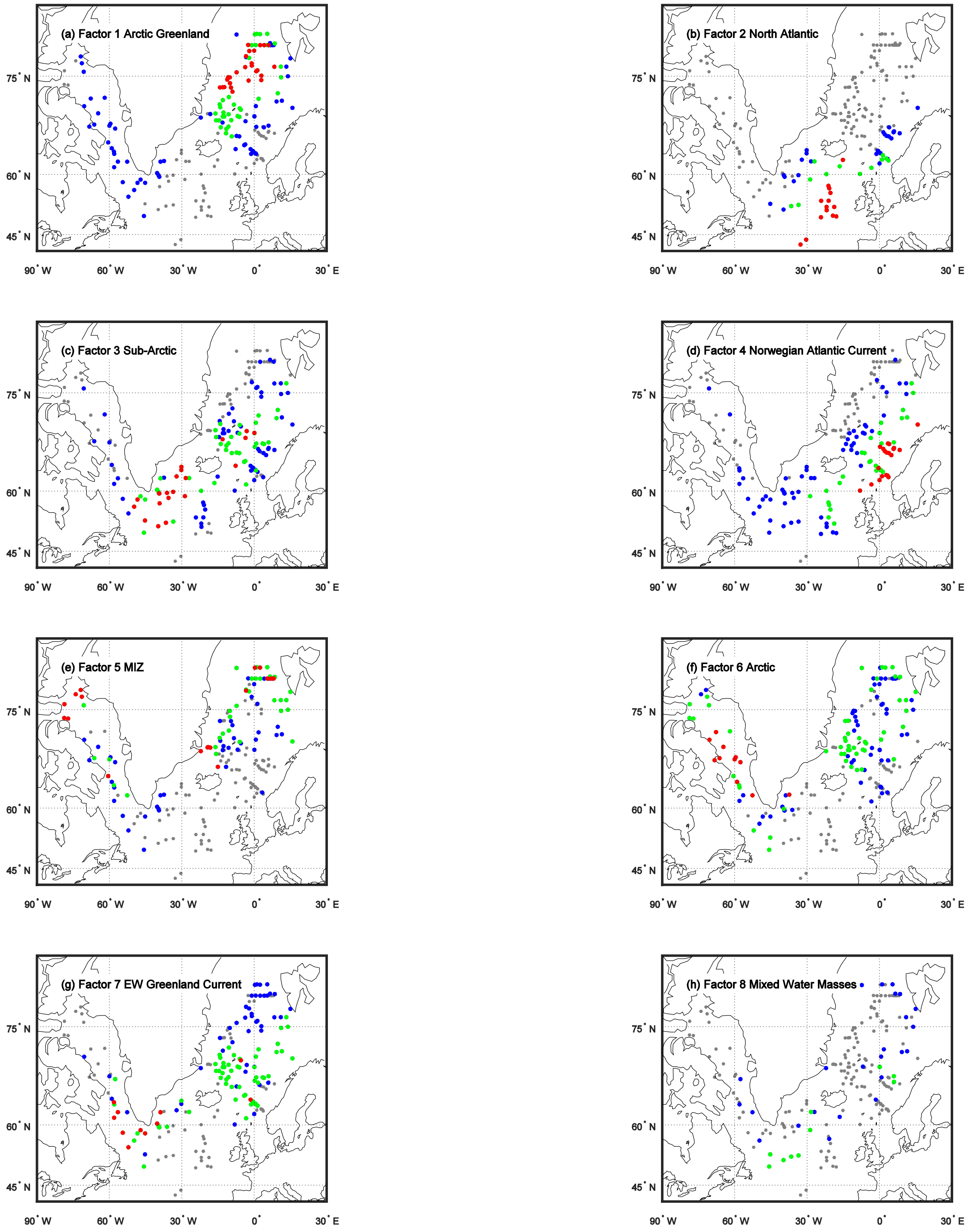

Supplementary Figure 1 | The geographical distribution of the derived factor loadings based on the modern calibration data set[1]. Colors represent factor loadings (>0.1=blue, >0.3=green and >0.7=red) for each factor assemblage; (a) the Greenland Arctic Water assemblage, (b) the North Atlantic Current assemblage, (c) the Subarctic Water assemblage, (d) the Norwegian Atlantic Current assemblage, (e) the Marginal Ice Zone assemblage, (f) the Arctic Water assemblage and (g) the East-West Greenland Current assemblage and (h) the Transitional Water assemblage. Some of the factor assemblages in this figure are presented according to the names and numbering in Andersen *et al.*[2].

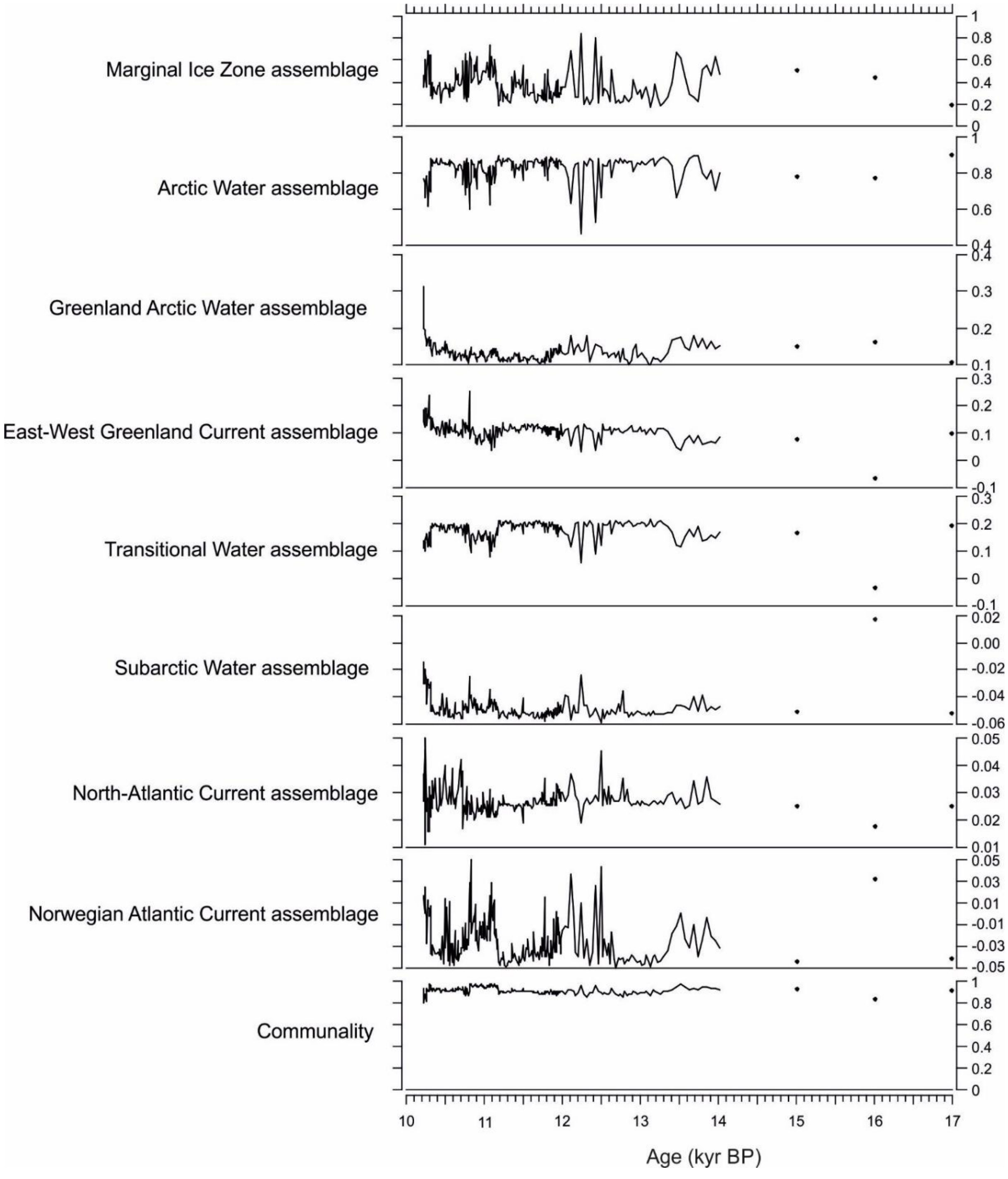

Supplementary Figure 2 | Factor loadings for core SL 170. Factor assemblages from the top; the Marginal Ice Zone, the Arctic Water, the Greenland Arctic Water, the East-West Greenland Current, the Transitional Water, the Subarctic Water, the North-Atlantic Current, the Norwegian Atlantic Current and communalities. Factors are scaled differently to make the smaller variations detectable. The assemblages are named after their relation to modern hydrography in Andersen et *al.*[2] with the exception of the Marginal Ice Zone assemblage that was re-named from its original name *Sea Ice* assemblage to better reflect the ecology of this group. The applied analysis resulted in high communalities (0.85-0.97, 0.91 on average) on the derived VARIMAX factor scores, which points to a good skill of the modern calibration data set for inferring the past hydrographic variations in the area in terms of the modern distribution of surface water masses in the northern North Atlantic.

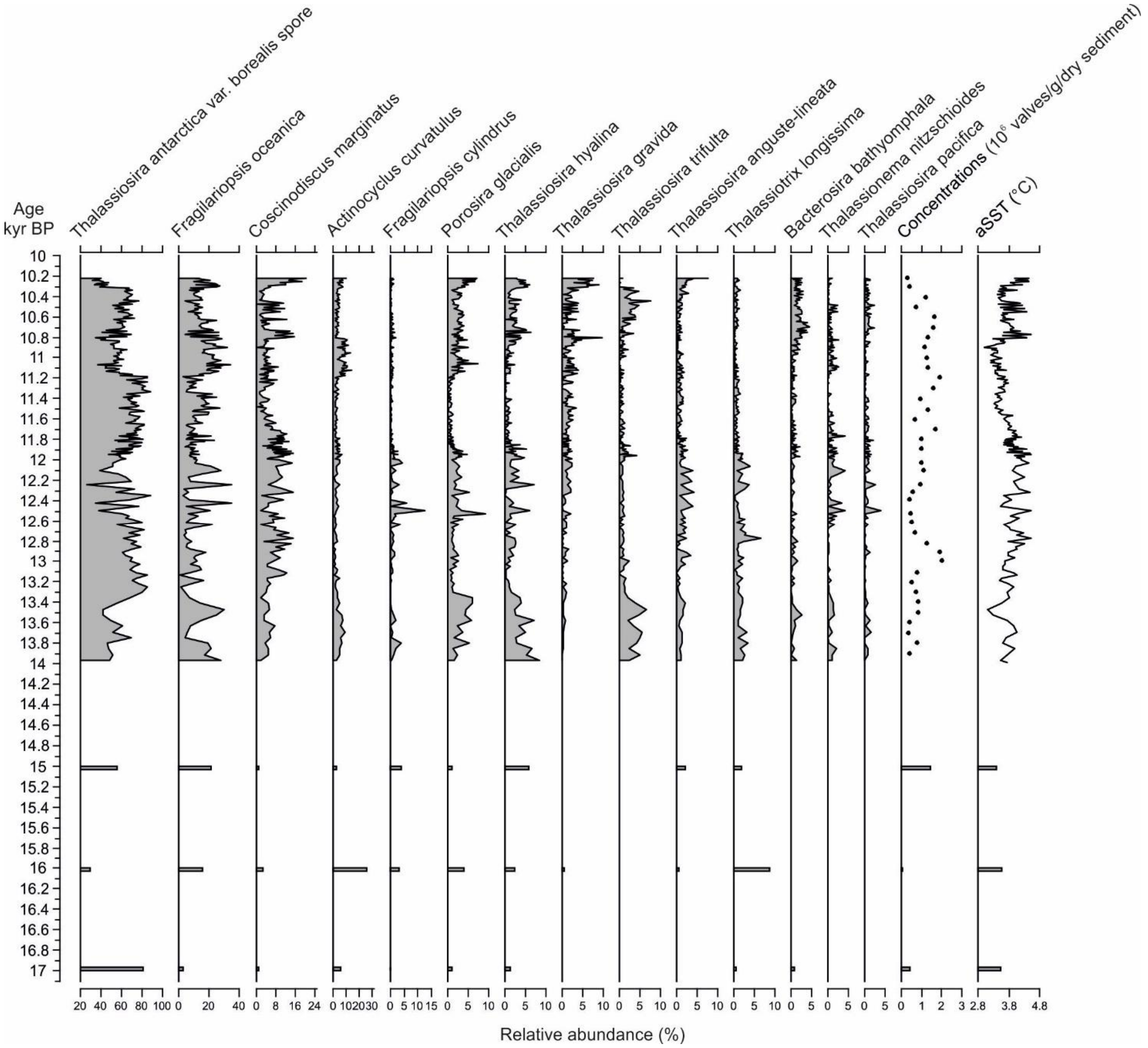

Supplementary Figure 3 | Relative abundances of the 14 most common diatom species (>3% in at least one sample) in core SL 170 including diatom concentrations and aSSTs. In total 40 diatom species were found in the record. The 14 most common species presented in the figure comprise 93.6-100 % (avg. 98.3%) of the total counts per sample. Additional three samples were analysed below the studied interval in order to crudely assess sea surface conditions prior to the time period this study focuses on. These samples represent the ages 15, 16 and 17 kyr BP based on our chronology. Reconstructed aSSTs for these samples are 3.4°C, 3.6°C and 3.5°C respectively, suggesting colder surface conditions prior to Younger Dryas.

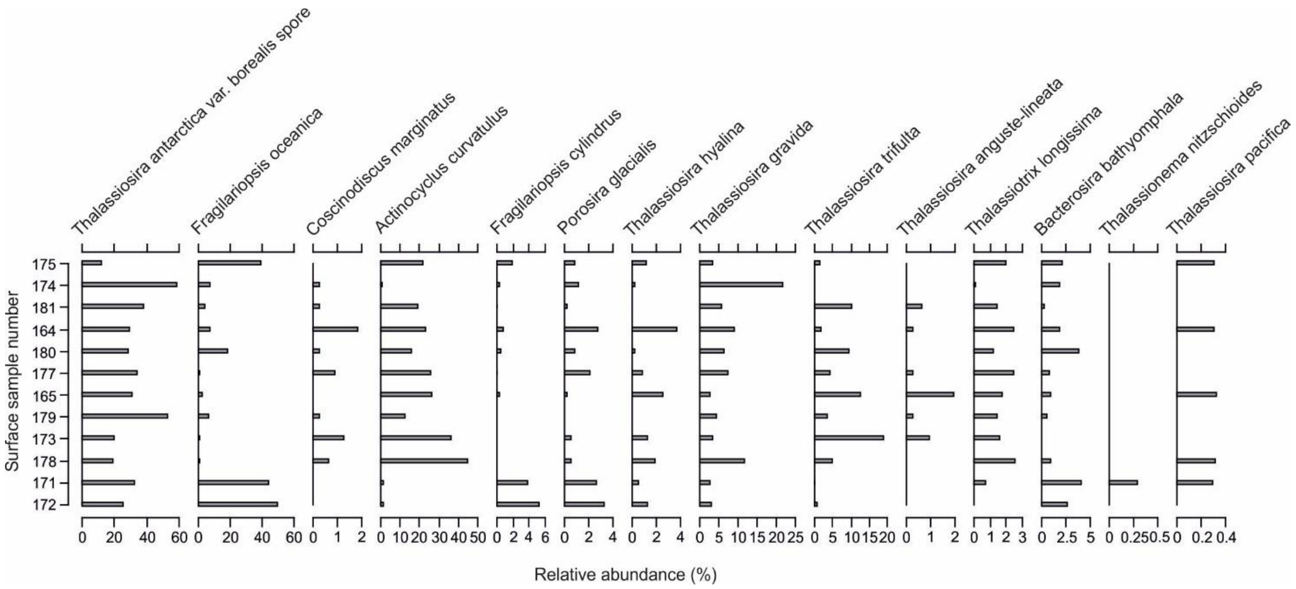

Supplementary Figure 4 | Relative abundances of the 14 most common diatom species found in core SL 170 in 12 surface sediment samples from the Baffin Bay region (65-75°N).

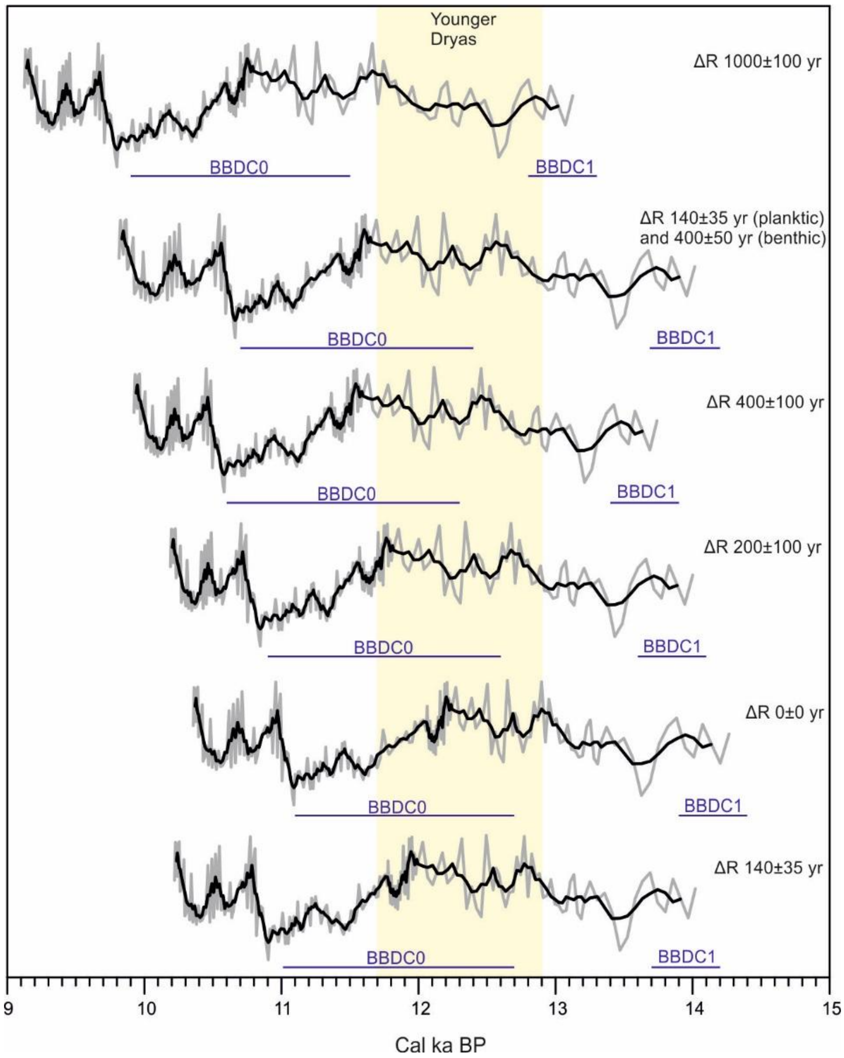

Supplementary Figure 5 | aSST record and the timing of the detrital carbonate events (BBDC0 and BBDC1)[15] plotted using different age-depth models. All models are derived by applying identical settings in the Bacon program[27] varying only the local reservoir correction age (ΔR). Age-depth models from bottom upwards; (1) ΔR=140±35 years (used in this study), (2) ΔR=0±0 years, (3) ΔR=200±100 years, (4) ΔR=400±100 years, (5) ΔR=variable (140±35 years for planktic foraminifera and mollusk fragments and 400±50 years for benthic foraminifera) and (6) ΔR=1000±100 years. The Younger Dryas period (12.9-11.7 kyr BP[28]) is marked with yellow shading.

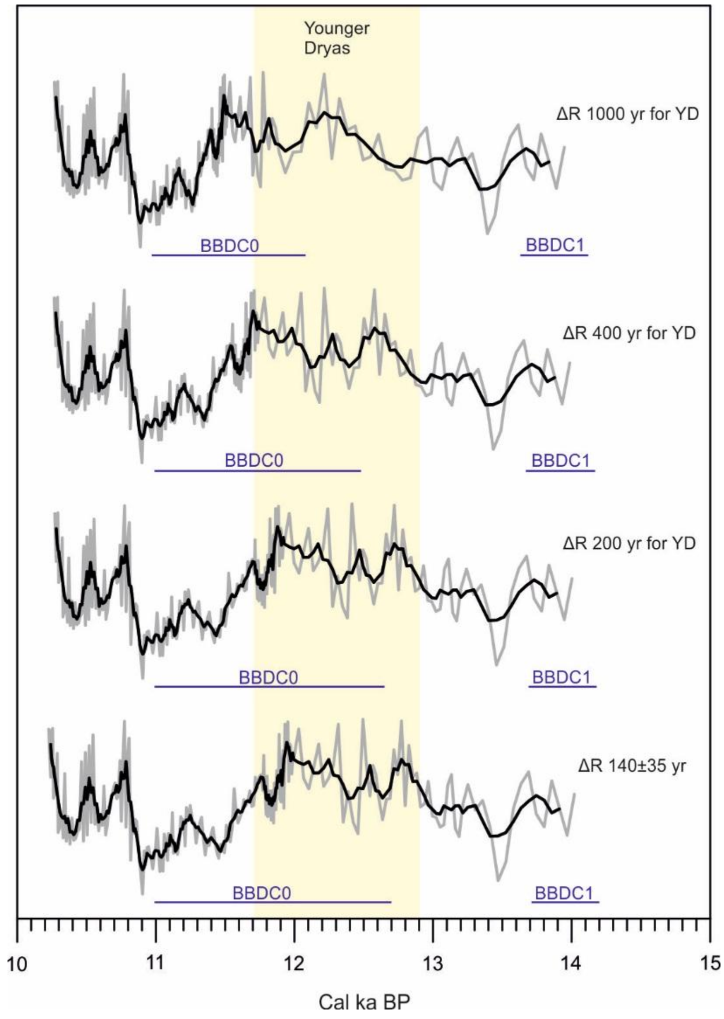

Supplementary Figure 6 | The aSST record and the timing of the detrital carbonate events (BBDC0 and BBDC1)[15] plotted using different age-depth models. All models are derived by applying identical settings in the Bacon program[27] varying only the local reservoir correction age (ΔR) within the YD period, while using the ΔR of 140±35 years for the other periods. Age-depth models from the bottom upwards; (1) constant ΔR=140±35 years (used in this study), (2) ΔR=200±100 years for the YD period, (3) ΔR=400±100 years for the YD period, (4) ΔR=1000±100 years for the YD period. The Younger Dryas period (12.9-11.7 kyr BP[28]) is marked with yellow shading.

Supplementary Table 1 | Factor loading and communalities for diatom assemblages in 12 surface sediment samples from the Baffin Bay region within 65-75°N[1].

Surface sample num.	Lat.	Lon.	Greenland Arctic Water	North Atlantic Current	Subarctic Water	Norwegian Atlantic Current	Marginal Ice Zone	Arctic Water	East-West Greenland Current	Transitional Water	Communality
175	66.00	-60.50	0.13	-0.01	0.05	0.05	0.82	0.33	-0.05	-0.12	0.82
174	68.23	-57.62	0.14	0.01	0	0.03	0.28	0.8	0.42	0.18	0.95
181	68.53	-68.33	0.24	0.01	0.09	0.04	0.21	0.92	0.08	0	0.97
164	68.67	-60.00	0.2	-0.01	0.11	0.07	0.3	0.86	0.15	-0.05	0.91
180	68.84	-66.27	0.23	0	0.11	0.05	0.55	0.75	0.1	-0.03	0.94
177	68.97	-59.57	0.2	0	0.08	0.06	0.14	0.92	0.1	-0.03	0.92
165	70.46	-64.66	0.29	0	0.06	0.05	0.16	0.88	-0.03	-0.06	0.89
179	71.43	-70.44	0.17	0.01	0.03	0	0.26	0.93	0.12	0.1	0.98
173	72.41	-67.72	0.3	-0.01	0.07	0.09	0.05	0.72	-0.11	-0.11	0.64
178	72.51	-61.96	0.19	-0.02	0.11	0.12	0.06	0.69	0.05	-0.17	0.57
171	74.02	-77.12	0.1	0.01	-0.01	-0.01	0.87	0.45	0.08	0.07	0.97
172	74.09	-78.72	0.09	0.01	0	-0.01	0.93	0.31	0.06	0.03	0.98

Supplementary Table 2 | The influence of ΔR correction ages on the chronology used in this study.

**Reservoir correction (ΔR)**	**0±0**	**200±100**	**400±100**	**1000±100**	**Variable (p=140±35 & b=400±50)**
Avg. shift (yrs)	174	94	325	1081	226
Age range (yrs)	118-286	0-209	247-415	872-1238	5-402

Supplementary References

1. Miettinen, A., Divine, D. V., Husum, K., Koç, N. & Jennings, A. Exceptional ocean surface conditions on the SE Greenland shelf during the Medieval Climate Anomaly. *Paleoceanography* 30, 1657-1674 (2015).

2. Andersen, C., Koç, N., Jennings, A. & Andrews, J. Nonuniform response of the major surface currents in the Nordic Seas to insolation forcing: Implications for the Holocene climate variability. *Paleoceanography* 19, PA2003 (2004).

3. Berner, K. S., Koç, N., Divine, D., Godtliebsen, F. & Moros, M. A decadal-scale Holocene sea surface temperature record from the subpolar North Atlantic constructed using diatoms and statistics and its relation to other climate parameters. *Paleoceanography* 23, PA2210 (2008).

4. Lloyd, J. *et al*. A 100 yr record of ocean temperature control on the stability of Jakobshavn Isbrae, West Greenland. *Geology* 39, 867-870 (2011).

5. Sheldon, C. *et al*. Ice stream retreat following the LGM and onset of the west Greenland current in Uummannaq Trough, west Greenland. *Quat. Sci. Rev.* 147, 27-46 (2016).

6. Jennings, A. E. *et al*. Paleoenvironments during Younger Dryas-Early Holocene retreat of the Greenland Ice Sheet from outer Disko Trough, central west Greenland. *J. Quat. Sci.* 29, 27-40 (2014).

7. Ouellet-Bernier, M., de Vernal, A., Hillaire-Marcel, C. & Moros, M. Paleoceanographic changes in the Disko Bugt area, West Greenland, during the Holocene. *Holocene* 24, 1573-1583 (2014).

8. Perner, K. *et al*. Centennial scale benthic foraminiferal record of late Holocene oceanographic variability in Disko Bugt, West Greenland. *Quat. Sci. Rev.* 30, 2815-2826 (2011).

9. Perner, K., Moros, M., Jennings, A., Lloyd, J. M. & Knudsen, K. L. Holocene palaeoceanographic evolution off West Greenland. *Holocene* 23, 374-387 (2013).

10. Hogan, K. A., Ó Cofaigh, C., Jennings, A. E., Dowdeswell, J. A. & Hiemstra, J. F. Deglaciation of a major palaeo-ice stream in Disko Trough, West Greenland. *Quat. Sci. Rev.* 147, 5-26 (2016).

11. Jennings, A. E. *et al*. Ocean forcing of Ice Sheet retreat in central west Greenland from LGM to the early Holocene. *Earth Planet. Sci. Lett.* 472, 1-13 (2017).

12. Gibb, O. T., Steinhauer, S., Frechette, B., de Vernal, A. & Hillaire-Marcel, C. Diachronous evolution of sea surface conditions in the Labrador Sea and Baffin Bay since the last deglaciation. *Holocene* 25, 1882-1897 (2015).

13. Knudsen, K. L., Stabell, B., Seidenkrantz, M., Eiríksson, J. & Blake, W., Jr. Deglacial and Holocene conditions in northernmost Baffin Bay: sediments, foraminifera, diatoms and stable isotopes. *Boreas* 37, 346-376 (2008).

14. Ledu, D., Rochon, A., de Vernal, A. & St-Onge, G. Holocene paleoceanography of the northwest passage, Canadian Arctic Archipelago. *Quat. Sci. Rev.* 29, 3468-3488 (2010).

15. Jackson, R. *et al*. Asynchronous instability of the North American-Arctic and Greenland ice sheets during the last deglaciation. *Quat. Sci. Rev.* 164, 140-153 (2017).

16. Jennings, A., Gronvold, K., Hilberman, R., Smith, M. & Hald, M. High-resolution study of Icelandic tephras in the Kangerlussuaq Trough, southeast Greenland, during the last deglaciation. *J. Quat. Sci.* 17, 747-757 (2002).

17. Knutz, P. C., Sicre, M., Ebbesen, H., Christiansen, S. & Kuijpers, A. Multiple-stage deglacial retreat of the southern Greenland Ice Sheet linked with Irminger Current warm water transport. *Paleoceanography* 26, PA3204 (2011).

18. Heinrich, H. Origin and Consequences of Cyclic Ice Rafting in the Northeast Atlantic-Ocean during the Past 130,000 Years. *Quatern. Res.* 29, 142-152 (1988).

19. Andrews, J., Kirby, M., Aksu, A., Barber, D. & Meese, D. Late Quaternary detrital carbonate (DC-) layers in Baffin Bay marine sediments (67 degrees-74 degrees N): Correlation with Heinrich events in the North Atlantic? *Quat. Sci. Rev.* 17, 1125-1137 (1998).

20. Andrews, J. T., Gibb, O. T., Jennings, A. E. & Simon, Q. Variations in the provenance of sediment from ice sheets surrounding Baffin Bay during MIS 2 and 3 and export to the Labrador Shelf Sea: site HU2008029-0008 Davis Strait. *J. Quat. Sci.* 29, 3-13 (2014).

21. Simon, Q., St-Onge, G. & Hillaire-Marcel, C. Late Quaternary chronostratigraphic framework of deep Baffin Bay glaciomarine sediments from high-resolution paleomagnetic data. *Geochem. Geophys. Geosyst.* 13, Q0AO03 (2012).

22. Simon, Q., Hillaire-Marcel, C., St-Onge, G. & Andrews, J. T. North-eastern Laurentide, western Greenland and southern Innuitian ice stream dynamics during the last glacial cycle. *J. Quat. Sci.* 29, 14-26 (2014).

23. Andrews, J. *et al*. A Heinrich-Like Event, H-0 (Dc-0) - Source(s) for Detrital Carbonate in the North-Atlantic during the Younger Dryas Chronozone. *Paleoceanography* 10, 943-952 (1995).

24. Andrews, J. *et al*. Abrupt changes in marine conditions, Sunneshine fiord, eastern Baffin island, NWT during the last deglacial transition: Younger Dryas and H-O events. *Geol. Soc. London, Spec. Publ.* 111, 11-27 (1996).

25. Jennings, A., Andrews, J., Pearce, C., Wilson, L. & Ólfasdóttir, S. Detrital carbonate peaks on the Labrador shelf, a 13-7 ka template for freshwater forcing from the Hudson Strait outlet of the Laurentide Ice Sheet into the subpolar gyre. *Quat. Sci. Rev.* 107, 62-80 (2015).

26. Lloyd, J., Park, L., Kuijpers, B. & Moros, M. Early holocene palaeoceanography and deglacial chronology of Disko Bugt, West Greenland. *Quat. Sci. Rev.* 24, 1741-1755 (2005).

27. Blaauw, M. & Christen, J. A. Flexible Paleoclimate Age-Depth Models Using an Autoregressive Gamma Process. *Bayesian Anal.* 6, 457-474 (2011).

28. Rasmussen, S. *et al*. A new Greenland ice core chronology for the last glacial termination. *J. Geophys. Res.* 111, D06102 (2006).
